# Parametric assumptions equate to hidden observations: comparing the efficiency of nonparametric and parametric models for estimating time to AIDS or death in a cohort of HIV-positive women

**DOI:** 10.1186/s12874-018-0605-8

**Published:** 2018-11-19

**Authors:** Jacqueline E. Rudolph, Stephen R. Cole, Jessie K. Edwards

**Affiliations:** Department of Epidemiology, University of North Carolina at Chapel Hill, 135 Dauer Drive, 2101 McGavran-Greenberg Hall, CB #7435, Chapel Hill, 27599 NC USA

**Keywords:** Survival analysis, Nonparametric model, Parametric model, Statistical efficiency

## Abstract

**Background:**

When conducting a survival analysis, researchers might consider two broad classes of models: nonparametric models and parametric models. While nonparametric models are more flexible because they make few assumptions regarding the shape of the data distribution, parametric models are more efficient. Here we sought to make concrete the difference in efficiency between these two model types using effective sample size.

**Methods:**

We compared cumulative risk of AIDS or death estimated using four survival models – nonparametric, generalized gamma, Weibull, and exponential – and data from 1164 HIV patients who were alive and AIDS-free in 1995. We added pseudo-observations to the sample until the spread of the 95% confidence limits for the nonparametric model became less than that for the parametric models.

**Results:**

We found the 3-parameter generalized gamma to be a good fit to the nonparametric risk curve, but the 1-parameter exponential both underestimated and overestimated the risk at different times. Using two year-risk as an example, we had to add 354, 593, and 3960 observations for the nonparametric model to be as efficient as the generalized gamma, Weibull, and exponential models, respectively.

**Conclusions:**

These added observations represent the hidden observations underlying the efficiency gained through parametric model form assumptions. If the model is correctly specified, the efficiency gain may be justified, as appeared to be the case for the generalized gamma model. Otherwise, precision will be improved, but at the cost of specification bias, as was the case for the exponential model.

**Electronic supplementary material:**

The online version of this article (10.1186/s12874-018-0605-8) contains supplementary material, which is available to authorized users.

## Background

When conducting survival analysis, a researcher might consider using either of two classes of models to describe time to the event of interest: parametric or nonparametric. In deciding between them, there is a well-known yet relatively under-discussed trade off. The researcher could choose the statistical efficiency and rigid model form assumptions of the parametric approach or the fewer model form assumptions but reduced efficiency of the nonparametric approach.

Suppose the researcher selects a finite-dimension parametric model, in which he first assumes the survival function follows a particular form and then estimates the parameters of that model [1]. The parameters allow the model to vary in constrained ways (e.g. in its location or scale) [2]. For example, he might have specified an exponential survival model, which constrains the estimated survival curve to be a function of a single parameter that governs the size of a constant hazard over the study period. However, for his estimates to be essentially unbiased, the data generating mechanism must coincide closely with the exponential model. In reality, though, we know complex epidemiologic and biomedical data are unlikely to follow simple parametric forms [3].

Suppose another researcher was unwilling to make such assumptions regarding model form; she could instead apply a nonparametric model. For example, she might choose a nonparametric Kaplan-Meier estimator of the survival function [4], where there are as many parameters as there are distinct event times. This model makes no assumptions regarding the distribution of event times, allowing the survival curve to take on any monotonically decreasing shape. While this flexibility is highly attractive in theory, in practice, the benefit of leaving the model unconstrained may be offset by the decrease in efficiency compared to a less flexible parametric model, resulting in wider confidence intervals than a parametric analog.

Here, we aim to make concrete the difference in efficiency between a nonparametric and three parametric survival models, by quantifying the number of hidden observations that the assumptions of the parametric models effectively add to our sample. Put another way, we determined the number of additional participants one would need to enroll if they wish to use a nonparametric model (thereby making fewer assumptions) but still have results be as efficient as a parametric model was at the original sample size. To demonstrate this, we used data from participants in the Women’s Interagency Human Immunodeficiency Virus (HIV) Study (WIHS) [5].

## Methods

Our study population was 1164 HIV-positive women enrolled in the WIHS who were alive and free of Acquired Immunodeficiency Syndrome (AIDS) on December 6, 1995 [6]. The women were followed until AIDS or death, loss to follow-up, or administrative censoring on September 28, 2006. In this sample, we estimated crude cumulative risk over the approximately 10 years of follow-up for a combined endpoint of mortality and clinical AIDS using four survival models (for more details, see Additional file 1). We first used the nonparametric Nelson Aalen estimator. We then compared the nonparametric model to three parametric models: 3-parameter generalized gamma, 2-parameter Weibull, and 1-parameter exponential. For all four models, pointwise upper and lower 95% confidence limits of the cumulative risk were obtained using the delta method [7, 8].

To conduct the comparison of the four models, we chose as an example the risk at year two. Our metric to compare the efficiency of the nonparametric and parametric models was the width of the 95% confidence limits (CL). Then, to take into consideration both bias and precision, we calculated the root mean square error (RMSE) for each model, assuming that the nonparametric risk was unbiased. We further took the average of the 95% CL width at each distinct event time across the entire 10 years of follow-up, as a way to compare the difference in statistical efficiency across the entire risk curve.

Finally, to determine the number of observations we would need to add to our sample for the nonparametric model to be as efficient as the parametric model at the starting sample size, we added pseudo-observations (or ghosts) one-by-one to the 1164 data points. To do this in a manner that did not perturb the shape of the function we distributed the ghosts equally across the data points (i.e., we assigned each original observation a weight that increased by 1/1164 every time a new pseudo-observation was added). Thus, for the original data set, the weight was one, and the weight was two when the sample had been doubled. After each observation was added, we repeated the nonparametric analysis until the CL difference for the weighted nonparametric model was smaller than that for the parametric model at the original sample size. All statistical analyses were carried out in SAS software version 9.4 (SAS Institute Inc., Cary, NC).

## Results

**Fig. 1:**
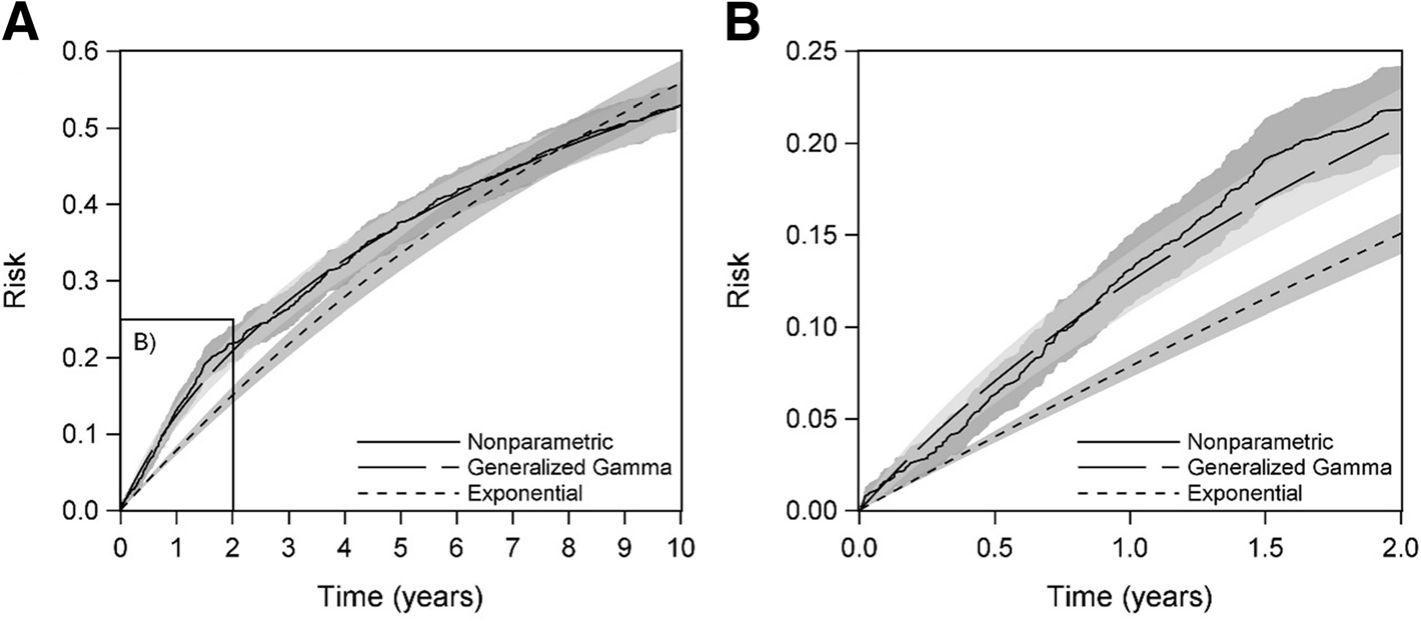
Cumulative risk of AIDS or death estimated using a nonparametric, generalized gamma, and exponential model and 95% confidence intervals (**a**) over all 10 years of follow-up and (**b**) through the first 2 years of the full follow-up

The cumulative risk curves across all 10 years of follow-up for three of the models are shown in Fig. 1a. The figure shows that the generalized gamma risk curve was a good fit to the data (given by the nonparametric curve), while the exponential curve was not. The latter model both underestimated (at earlier time points) and overestimated (at later time points) the risk estimated nonparametrically. Also, as expected, the nonparametric model had the widest point-wise confidence intervals and the exponential model had the narrowest confidence intervals. The average 95% CL width at each distinct event time concurred with what was seen visually. As can be seen in Table 1, the nonparametric model had the largest average CL width, followed in order by the generalized gamma, Weibull, and exponential models.

**Table 1:** Estimated risks and model efficiency by the number of observations added to the sample

Approach	N	M	Risk^a^	95% CLs^a^	CL Difference^a^	Average CL Difference^b^
Nonparametric	1164	0	0.218	0.194, 0.242	0.048	0.047
Generalized Gamma	1164	0	0.210	0.189, 0.231	0.042	0.042
Nonparametric	1164	354	0.218	0.197, 0.239	0.042	0.041
Weibull	1164	0	0.200	0.180, 0.219	0.039	0.040
Nonparametric	1164	593	0.218	0.199, 0.238	0.039	0.038
Exponential	1164	0	0.152	0.141, 0.163	0.023	0.030
Nonparametric	1164	3960	0.218	0.207, 0.230	0.023	0.022

*Abbreviations*, *N* sample size, *M* pseudo-observations added, *CL* confidence limit

^a^At two years

^b^At all event times

Comparing the estimated two-year risk of AIDS or death, 95% CLs, and CL difference of all four models at the starting sample size (M = 0) and when the nonparametric model became as efficient as the parametric generalized gamma (M = 354), Weibull (M = 593), and exponential (M = 3960) models. The average 95% CL difference at all event times is also given

These results were particularly evident at two years (Fig. 1b). The two-year risk of AIDS or death in the 1164 WIHS participants estimated using the nonparametric model was 0.22 (95% CL difference: 0.048; RMSE: 0.078). For the generalized gamma model, the risk was 0.21 (95% CL difference: 0.042; RMSE: 0.074), and for the exponential model, the risk was 0.15 (95% CL difference: 0.023; RMSE: 0.086). For the Weibull model, the risk was 0.20 (95% CL difference: 0.039; RMSE: 0.073). Thus, the generalized gamma approximated the nonparametric risk well but was more precise; the exponential model was highly precise but biased. The Weibull model sat between these two extremes in terms of bias and precision, resulting in a smaller root-MSE than either of the other parametric models.

We found that we would need to add 354 pseudo-observations for the nonparametric two-year risk to become as efficient as the generalized gamma model, 593 pseudo-observations for the nonparametric two-year risk to become as efficient as the Weibull model, and 3960 pseudo-observations for it to become as efficient as the exponential. These results are summarized in Table 1.

## Discussion

Parametric models are always more efficient than nonparametric models, and, in this demonstration, we expressed that precision difference in terms of a difference in effective sample size. We found that we had to add 354 observations to our sample for the two-year risk of AIDS or death estimated nonparametrically to become as efficient as the risk estimated in the original sample of WIHS participants using a generalized gamma model. If we compared to an exponential model, though, we would have needed to more than quadruple the sample (an increase from 1164 to 5124 participants) to have an equally efficient nonparametric model. While this indicated that the exponential model was far more efficient than either the nonparametric or the generalized gamma model, examination of the risk curves revealed that it also greatly underestimated the risk at two years. If we had chosen the exponential model to estimate our 10-year risk curves, we would have been very precise but biased.

The difference in efficiency between parametric and nonparametric models has previously been described in various ways. First, parametric models are by definition smooth curves through the data, the form depending upon the shape assumption chosen. The smoothness arises as a result of “borrowing” information from all observations. For instance, predicted values of the model are determined based on observed data near a focal point as well as data observed distally [9]. One can envision this smoothing process as using each observed data point’s information more than once. This borrowing of information increases model efficiency and was mimicked in our study by adding the pseudo-observations to the nonparametric model’s sample. Nonparametric models, on the other hand, are less efficient than parametric models because they do not borrow information. Nonparametric models work only at the focal point and have as many parameters as there are distinct data points.

Second, the difference in precision between parametric and nonparametric models has been described in terms of “approximation error” [10]. Parametric models presume the model form to be correct and thus do not account for any error that arises because the model’s estimate is only an approximation of the truth. This supposed knowledge of the true specification adds information beyond the data (again represented here by the pseudo-observations). Parametric models are therefore more efficient than nonparametric models (which make no such assumptions) with the same number of observations.

When the parametric model happens to be correctly specified, the hidden observations might be seen as a benefit (i.e. an assumption correctly leveraged). In such a setting, one has a model that is both unbiased (assuming no other biases) and efficient. However, in real life, parametric models are rarely perfectly specified [3]. If the difference in efficiency between the nonparametric and parametric model is great enough and if their point estimates also differ, two researchers could arrive at different inferences [10]. In these situations, the hidden observations, representing the additional information gained through the model form assumptions, provide one with a result that is precise but potentially biased. For instance, one could easily see a researcher who used the highly efficient but biased exponential model from our example arriving at a different conclusion about the two-year risk of mortality or AIDS in this WIHS population than a researcher using the nonparametric model.

There were several limitations of our demonstration. For one, the results were meant to be illustrative and, while the idea generalizes, the numerical results cannot be generalized to other situations. The number of ghosts hidden by the constraints of a given parametric model will likely be context-dependent, being based on factors such as starting sample size, the underlying data generation distribution, and the assumed parametric form (e.g. the number of parameters), among others. For instance, our results would have differed if we had selected the ten-year risk to compare. Additionally, we were not attempting to determine the number of observations that had to be added for the nonparametric model to become as smooth as the corresponding parametric model. Such continuity would require an infinite number of data points. Instead, we used the CL difference at one point along the cumulative risk curve as a working approximation for model efficiency and determined the finite number of observations that had to be added for the nonparametric risk to become more efficient than the parametric risk.

## Conclusions

Here, we made concrete the difference in precision between a nonparametric model and three corresponding parametric models. We have shown that the efficiency gain resulting from the parametric form constraints can be viewed as similar to working in a sample that contains a number of hidden observations or “ghosts.” In the most extreme case, we saw that the exponential model’s strict one-parameter form restriction was equivalent to almost 4000 additional observations but that, if we used that highly precise exponential model to estimate the two-year risk of death or AIDS, we would obtain a precise but biased answer.

The question remains, then, which model should we choose in practice. On the one hand, we might always select the nonparametric model to limit the number of assumptions we are making, which is theoretically appealing. On the other, small sample sizes or the desire to include many variables in one’s model routinely make nonparametric models infeasible, particularly if one is unable to add more observations to one’s sample. In such cases, a parametric model may be needed, but one should always consider the potential for bias due to model misspecification. Additionally, while not explored here, a middle ground does exist in semiparametric models. Semiparametric models decompose the parameter vector into a parametric and nonparametric components; thus, they are more efficient than nonparametric models but require fewer assumptions than parametric models [11].

Which model makes the best choice will no doubt be context- and data-dependent, and the decision process will most likely include consideration of the bias/variance trade-off. Our work seeks to remind those making such decisions that the efficiency gained from a parametric model is never “free” but can rather be directly related to a certain number of pseudo-observations closely tied to a chosen (and assumed correct) parametric model specification.

## Additional file

Additional file 1: Survival formulas. (PDF 534 kb)

## References

[1] Cole SR, Chu H, Greenland S (2014). Maximum likelihood, profile likelihood, and penalized likelihood: a primer. Am J Epidemiol.

[2] Casella G, Berger RL (2002). Statistical inference.

[3] van der Laan MJ (2011). Rose S. targeted learning causal inference for observational and experimental data.

[4] Kaplan EL, Meier P (1958). Nonparametric-estimation from incomplete observations. J Am Stat Assoc.

[5] Bacon MC, von Wyl V, Alden C, Sharp G, Robison E, Hessol N (2005). The Women's interagency HIV study: an observational cohort brings clinical sciences to the bench. Clin Diagn Lab Immunol.

[6] Lau B, Cole SR, Gange SJ (2009). Competing risk regression models for epidemiologic data. Am J Epidemiol.

[7] Delta Method CC (1998). Encyclopedia of biostatistics.

[8] SAS Institute I. The NLMIXED Procedure: Prediction 2015 [Available from: http://support.sas.com/documentation/cdl/en/statug/68162/HTML/default/viewer.htm#statug_nlmixed_details33.htm.

[9] Yatchew A (2003). Semiparametric regression for the applied econometrician.

[10] Horowitz J (2009). Semiparametric and nonparametric methods in econometrics.

[11] Tsiatis AA (2006). Semiparametric Theory and Missing Data.

