# Correction to: Parametric assumptions equate to hidden observations: comparing the efficiency of nonparametric and parametric models for estimating time to AIDS or death in a cohort of HIV-positive women

**DOI:** 10.1186/s12874-019-0691-2

**Published:** 2019-03-15

**Authors:** Jacqueline E. Rudolph, Stephen R. Cole, Jessie K. Edwards

**Affiliations:** Department of Epidemiology, University of North Carolina at Chapel Hill, 135 Dauer Drive, 2101 McGavran-Greenberg Hall, CB #7435, Chapel Hill, NC 27599 USA

**Correction to: BMC Medical Research Methodology (2018) 18:142**

10.1186/s12874-018-0605-8

In the original publication of this article [1], the numbers of the root mean square errors in the second paragraph of the result section are wrong. Other results and interpretation remain unchanged. The numbers are revised in the paragraph below:

These results were particularly evident at two years (Fig. 1b). The two-year risk of AIDS or death in the 1164 WIHS participants estimated using the nonparametric model was 0.22 (95% CL difference: 0.048; RMSE: 0.012). For the generalized gamma model, the risk was 0.21 (95% CL difference: 0.042; RMSE: 0.014), and for the exponential model, the risk was 0.15 (95% CL difference: 0.023; RMSE: 0.067). For the Weibull model, the risk was 0.20 (95% CL difference: 0.039; RMSE: 0.021). Thus, the generalized gamma approximated the nonparametric risk well but was more precise; the exponential model was highly precise but biased. The Weibull model sat between these two extremes in terms of bias and precision.
