# Supplementary material for: Parametric assumptions equate to hidden observations: comparing the efficiency of nonparametric and parametric models for estimating time to AIDS or death in a cohort of HIV-positive women
Source: BMC Med Res Methodol. 2018 Nov 19;18:142. doi: 10.1186/s12874-018-0605-8 (PMC6245810; doi:10.1186/s12874-018-0605-8)
Supplement: Supplementary file 1 — Survival formulas. (PDF 534 kb) [file 12874_2018_605_MOESM1_ESM.pdf]

Additional File 1. Survival formulas

To estimate survival to time t nonparametrically, we used the Nelson-Aalen survival estimator:

$$\hat{S}(t) = \exp\left[-\widehat{\mathrm{A}}(t)\right],$$

where $\widehat{\mathrm{A}}(t)$ is the cumulative hazard, $\widehat{\mathrm{A}}(t) = \sum_{t_j \le t} d_j/n_j$ and d_j is the number of events and n_j the number of individuals at risk just prior to time t_j (12).

We used three parametric survival estimators. The form of the 3-parameter generalized gamma survival function is given below:

$$S(t) = \begin{cases} \text{if } \lambda > 0 & 1 - \Gamma\left[\lambda^{-2}\left(e^{-\beta}t\right)^{\frac{\lambda}{\sigma}}; \lambda^{-2}\right] \\ \text{if } \lambda < 0 & \Gamma\left[\lambda^{-2}\left(e^{-\beta}t\right)^{\frac{\lambda}{\sigma}}; \lambda^{-2}\right] \end{cases},$$

where $\Gamma(t;\gamma) = \int_0^t u^{\gamma-1}e^{-u}du/\Gamma(\gamma)$ is the distribution function for a gamma distribution (i.e., the incomplete gamma function), and $\Gamma(\gamma) = \int_0^\infty u^{\gamma-1}e^{-u}du$ is the complete gamma function (13). The generalized gamma's three parameters govern the model's location ($\beta > 0$), scale ($\sigma > 0$), and shape ($\lambda > 0$).

The 2-parameter Weibull survival function takes the form:

$$S(t) = \exp[-\lambda t^{\gamma}],$$

where λ is the scale parameter and γ the shape parameter. Note, that the λ here differs from the one above, but we have switched parameterization to align with convention. The Weibull model can be considered a special case of the generalized gamma that occurs when $\lambda = 1$ (using the different parameterization from the generalized gamma above).

Finally, the 1-parameter exponential survival formula takes the form:

$$S(t) = \exp[-\lambda t],$$

where λ is the scale parameter (14). It is easy to see that this is simply the Weibull model where one assumes the shape parameter is equal to one.

For all three models, we used survival to obtain the risk: $R(t) = 1 - S(t)$.

References

12. Borgan Ø. Nelson-Aalen estimator. Encyclopedia of Biostatistics. New York: John Wiley; 1998. p. 2967-72.

13. Cox C, Chu H, Schneider MF, Munoz A. Parametric survival analysis and taxonomy of hazard functions for the generalized gamma distribution. Stat Med. 2007;26(23):4352-74.

14. Klein JP, Moeschberger ML. Survival Analysis: Techniques for Censored and Truncated Data. 2 ed. New York: Springer-Verlag; 2003. p. 37.
